# Humanized mouse models in MDS

**DOI:** 10.1038/s41419-025-07861-0

**Published:** 2025-07-17

**Authors:** Raluca Munteanu, Diana Gulei, Cristian Silviu Moldovan, Emanuele Azzoni, Laura Belver, Richard Feder, Simina Pirv, Anca Dana Buzoianu, Hermann Einsele, Moshe Mittelman, Gabriel Ghiaur, Robert Hasserjian, Ciprian Tomuleasa

**Affiliations:** 1 Department of Personalized Medicine and Rare Diseases, Medfuture Institute for Biomedical Research, Iuliu Hațieganu University of Medicine and Pharmacy, Cluj-Napoca, Romania; 2 Department of Nanosciences, Medfuture Institute for Biomedical Research, Iuliu Hațieganu University of Medicine and Pharmacy, Cluj-Napoca, Romania; 3 School of Medicine and Surgery, University of Milano-Bicocca, Monza, Italy; 4 Fondazione IRCCS San Gerardo dei Tintori, Monza, Italy; 5 Josep Carreras Leukaemia Research Institute, Badalona, Barcelona, Spain; 6 Catalan Institute of Oncology, Cancer Therapeutic Resistance Program (ProCURE), Badalona, Barcelona, Spain; 7 Medizinische Klinik Und Poliklinik II, Universitätsklinikum Würzburg, Würzburg, Germany; 8 Department of Hematology, Tel Aviv Sourasky Medical Center, Tel Aviv, Israel; 9 Department of Leukemia, Sidney Kimmel Cancer Center at Johns Hopkins, Johns Hopkins University School of Medicine, Baltimore, MD USA; 10 Department of Pathology, Massachusetts General Hospital—Harvard Medical School, Boston, MA USA; 11 Department of Hematology, Ion Chiricuta Oncology Institute, Cluj-Napoca, Romania; 12 Department of Hematology, Iuliu Hațieganu University of Medicine and Pharmacy, Cluj-Napoca, Romania

**Keywords:** Experimental models of disease, Preclinical research

## Abstract

Myelodysplastic syndromes (MDS) are heterogeneous hematopoietic stem cell disorders defined by ineffective hematopoiesis, multilineage dysplasia, and risk of progression to acute myeloid leukemia. Improvements have been made to identify recurrent genetic mutations and their functional roles, but translating this into preclinical models is still difficult. Traditional murine systems lack the human-specific cytokine support and microenvironmental support that is necessary to reproduce MDS pathophysiology. Humanized mouse models, particularly those incorporating human cytokines (e.g., MISTRG, NSG-SGM3, NOG-EXL), immunodeficient backgrounds, and co-transplantation strategies, have improved the engraftment and differentiation of human hematopoietic stem and progenitor cells. These models allow the study of clonal evolution, mutation-specific disease dynamics, and response to therapies in vivo. However, difficulties persist, such as limited long-term engraftment, incomplete immune reconstruction, and limited possibilities of modeling early-stage or low-risk MDS. This review presents an overview of current humanized and genetically engineered mouse models suitable for studying MDS, evaluating their capacity to replicate disease complexity, preserve clonal architecture, and support translational research. We highlight the need to develop new approaches to improve the actual methodologies and propose future directions for standardization and improved clinical relevance.

## Facts

- Conventional murine models lack the human cytokine support required for efficient engraftment and differentiation of MDS stem and progenitor cells.
- Cytokine-humanized strains support improved multilineage hematopoiesis but show limitations in long-term stability and lineage-specific maturation.
- There is no current murine model representative for MDS that fully copies the complex human bone marrow environment or immune system.

## Open questions

- How accurately do current humanized models reflect the sequential acquisition of mutations and clonal evolution in MDS?
- Can preclinical models be standardized for therapeutic evaluation to improve reproducibility and inter-study comparability?
- What are the optimal conditions (mouse strain, cytokine profile, preconditioning) for stable and representative engraftment of MDS?
- What role do underrepresented mutations (e.g., BCOR, STAG2) play in disease progression and therapy response, and how can these be integrated into future models?

## Introduction

Myelodysplastic syndromes (MDS) are a heterogeneous group of clonal hematopoietic bone marrow disorders characterized by abnormal hematopoiesis and the presence of dysplasia in one or more blood cell lineages, which have the potential to progress to acute myeloid leukemia (AML). Complex and less predictive interactions between genetic, epigenetic and microenvironmental factors contribute to the pathogenesis of MDS, making the development of animal models more challenging compared to AML, which is a more aggressive and well-defined disease [1]. The absence of sufficiently reliable MDS animal models has delayed the advancement of therapies. However, recent efforts have led to the improvement of more dependable and efficient animal models that better capitulate MDS providing a more optimistic perspective for future progress.

Early efforts to model hematologic malignancies focused on subcutaneous implantation of patient-derived AML cells into immunodeficient mice, as documented by early studies [2]. Functional engraftment of MDS cells in immunodeficient mice was only demonstrated in the early 2000s [3, 4]. However, these models had low efficiency and incomplete clonal representation. Later advancements like systemic engraftment via tail vein injections and the use of more immunocompromised strains (e.g., NSG, NOG mice lacking functional B, T, NK cells), improved hematological diseases representation in vivo [5].

Another breakthrough was the introduction of co-transplantation of MSCs with hematopoietic cells from MDS patients into immunocompromised mice which has shown mixed results, but while some studies report stable engraftment of mutations like *RUNX1* and *SF3B1*, others find MSCs provide only temporary support [6–9]. Also, preconditioning strategies, such as low-dose radiation or macrophage-depleting drugs like clodronate, help improve these models, particularly for low-risk MDS cases [10].

Humanized mouse models have revolutionized MDS research by facilitating the study of clonal dynamics and bone marrow microenvironment (BME) interactions in vivo. While early models relied on immunodeficient strains like NSG (NOD/SCID-γ) for basic engraftment, modern approaches such as cytokine-humanized mice (e.g., MISTRG) and genetically engineered HSPCs have improved MDS representation. For example, MISTRG mice, which express human M-CSF, IL-3, GM-CSF, SIRPα, and thrombopoietin, has substantial myeloid engraftment ( > 80% CD33+ cells) and preserve patient-specific mutations, exceeding traditional NSG models [6, 11–13].

Spontaneous MDS have the advantage of increased clonal heterogeneity, but generating such models require maintaining approximately 30,000 mice for 20 months, as reported by Zhou et al., making large-scale or long-term studies financially impractical [14].

## Mutations in MDS and their representation in preclinical mouse models

MDS are caused by various somatic mutations that disrupt critical regulatory processes controlling hematopoietic stem cell (HSC) self-renewal, differentiation, and apoptosis pathways [15]. High-throughput sequencing studies have shown recurrent mutations in genes involved in RNA splicing (*SF3B1, SRSF2, U2AF1, ZRSR2*), epigenetic regulation (*TET2, DNMT3A, ASXL1, EZH2*), transcriptional control (*RUNX1, TP53*), and signal transduction (RAS pathway genes, *CBL, JAK2*) [16–21]. The functional impact of these mutations varies, some of them promote early clonal hematopoiesis, and others facilitate disease progression and leukemic transformation.

For example, *SF3B1* mutations induce impaired erythropoiesis and the production of ring sideroblasts, while TP53 loss-of-function mutations correlate with genomic instability and resistance to therapy [22]. Table 1 provides an overview of MDS-associated mutations (selected based on their high frequency), their pathogenic mechanisms, occurrence in MDS patients, representative preclinical models, and associated limitations.

**Table 1:** MDS mutations: pathogenic mechanisms, frequency, preclinical models, and limitations.

Mutation	Pathological mechanism	Frequency in MDS patients	Preclinical models	Limitations
SF3B1K700E [22]	Defective RNA splicing, abnormal erythropoiesis, ring sideroblast formation SF3B1 mutations, including K700E, activate the NMD pathway	20–30% overall in MDS, but higher (up to 66%) in MDS with ring sideroblasts (MDS-RS) [92]	GEMM (SF3B1K700E knock-in); PDX (MISTRG6kitW41).	Murine erythropoiesis differences and progressive anemia (ring sideroblast formation incomplete) [93]. Species-specific differences in gene expression and regulation. Requires exogenous human cytokine to support MDS cell lines engraftment [82]. Differences in engraftment rates between patient samples [94]. Limited longevity -may not fully replicate long term disease and progression.
SRSF2P95H	Defective pre-mRNA splicing, leading to modifications in multiple RNA processing and splicing proteins, including HNRNPA2B1 splice alteration which disrupts hematopoietic differentiation in vivo [95]	14.6% [96]; 11.5% and 39.8% in patients with MDS and CMML [97]; 12% [98]	GEMM (SRSF2P95H Knock-in); Inducible Mx1-Cre Srsf2P95H/WT.	Species-specific differences in specific pathways and responses to the SRSF2 mutation. Recombination can occur without plpC, especially during transplantation procedures or in the presence of activated oncogenes [99]. pIpC injection can cause transient changes in HSPCs phenotypes and frequencies [100].
TP53 mutations	Loss of p53-mediated apoptosis, genomic instability, therapy resistance; associated with high risk MDS and poor prognosis	5–10% in de novo cases of MDS; 30–40% in therapy related MDS [101, 102]	GEMM (TP53-R172H Knock-in) [103]; PDX (NSG-SGM3, patient-derived clonal cells).	Mice exhibit MDS-like features but rarely progress to advanced stages without additional genetic alterations; single TP53 mutation alone is insufficient to fully replicate human MDS pathogenesis [104].
TET2 and DNMT3A loss	Impaired DNA methylation and hematopoietic differentiation, promoting clonal dominance. TET2 mutations reduce 5-hmC levels, stimulating HSC self-renewal and myeloid expansion; DNMT3A mutations cause hypomethylation due to reduced methyltransferase activity [104]	20–30% TET mutations, 8% DNMT3A, higher prevalence in older patients [104]	GEMM (TET2/DNMT3A double KO) [105, 106]; PDX (NSG-SGM3, patient-derived clonal cells) [107].	*TET2* mutations promote myeloid differentiation in committed progenitors. *DNMT3A* loss promotes unlimited HSC self-renewal [108].
NRAS/KRAS mutations	NRAS mutations activate the MEK/ERK pathway, promoting cell growth, survival, and inhibiting apoptosis in preleukemic cells. The Nras^G12D^ oncoprotein suppresses apoptosis in Cbfβ-SMMHC-expressing cells via MEK/ERK activation [109].	Mutations in KRAS/NRAS have been observed in MDS patients, but with varied frequencies depending on the study and MDS subtype [110]	GEMM (*Mx1-Cre, LSL-Nras*^*G12D*^ Knock-in) [111]; PDX (NSG-SGM3, patient-derived clonal cells).	Mx1-Cre system can exhibit spontaneous recombination without induction May not fully recapitulate the human bone marrow microenvironment, potentially affecting disease manifestation [99].
NUP98-HOXD13 fusion	NUP98-HOXD13 fusion inhibits megakaryocytic differentiation, increases apoptosis [112]	Reported in 0.4% of MDS cases [113]	GEMM (NUP98-HOXD13-Tg) [91]; PDX.	Rapid progression to AML. Limited availability of PDX models.
ASXL1	ASXL1 is a tumor suppressor gene and epigenetic regulator which encodes a protein that is part of DNA- or histone modifying complexes [114]	Mutations frequency in patients with MDS vary between 11 and 21% based on multiple reports [115]	GEMM (ASXL1-mutant mouse model; strains used: - C57BL/6-Ly5.1 (B6.SJL-Ptprc Pep3/BoyJ) (donor) - C57BL/6-Ly5.2 (wild-type B6) (recipient).	Long disease latency ( ~ 400 days). Lacks additional mutations, Incomplete replication of human MDS features, such as ineffective hematopoiesis and clonal evolution. Leukemia progression is inconsistent [116].
RUNX1	Impaired hematopoietic differentiation and increased self-renewal; mutations disrupt transcriptional regulation, promoting leukemogenesis. Loss of RUNX1 function causes inhibition of differentiation of HSCs, genomic instability, leading to increased DNA damage and impaired DNA repair. RUNX1 mutations may attenuate the G1-S phase and promote the proliferation of hematopoietic cells that occur during the mitotic phase of the cell cycle (G2/M) [117]	~10% (Higher in high-risk MDS and AML transformation). RUNX1 mutations account for about 10-15% of all somatic mutations detected in MDS. Mutated RUNX1 was identified as the main molecular predictor of rapid progression in LR-MDS. RUNX1 mutations present in 64% of cases [117, 118]	GEMM (RUNX1 Knock-in, Runx1ΔE9); PDX (NSG-SGM3, patient-derived clonal cells).	PDX models show variability in engraftment success, in particular with low-risk MDS samples GEMM models show delayed disease onset and incomplete phenotypic overlap with human MDS. Limited multilineage differentiation [25].
U2AF1	Disrupted RNA splicing causes abnormal exon recognition, impaired hematopoiesis, and clonal dominance. U2AF1 mutations affect splicing patterns, that impair normal hematopoietic function and promote clonal proliferation [119]	~8–11% (More common in higher-risk MDS [120]	GEMM (U2AF1S34F Knock-in, Mx1-Cre) [121]; PDX (NSG-SGM3, patient-derived clonal cells).	- Incomplete MDS phenotype; mice exhibit cytopenia and anemia but rarely progress to MDS/AML without secondary mutations. - Requires additional mutations (e.g., Runx1 loss) for leukemia development. - Limited overlap of splicing alterations with human MDS ( ~ 20%) [121]. Low engraftment rates, altered clonal composition, limited lifespan, microenvironment differences, and variable drug responses [121].
EZH2	Loss-of-function mutations in EZH2, a key component of the Polycomb Repressive Complex 2 (PRC2), lead to reduced trimethylation of histone H3 at lysine 27 (H3K27me3). EZH2 mutations may disrupt B cell development and lymphopoiesis [122]	~3–13% of MDS patients [123].	GEMM (MxT;Ezh2Δ/Δ; NHD13 [124]; PDX NOD/SCID/IL2rγ^−/−^ (NSG) [9].	In the NHD13 mouse model, Ezh2 loss minimally accelerated leukemia development [124].

Overview of the most common somatic mutations observed in MDS, including their biological impact, prevalence, associated mouse models, and translational limitations.

Expanding long-term HSCs in vitro is challenging, and the availability of MDS cell lines is limited, highlighting the necessity for in vivo models that accurately reproduce primary MDS [23]. The extent to which preclinical models replicate human MDS is an important consideration, particularly regarding hematopoietic microenvironment interactions and immunological regulation [24].

**Fig. 1:**
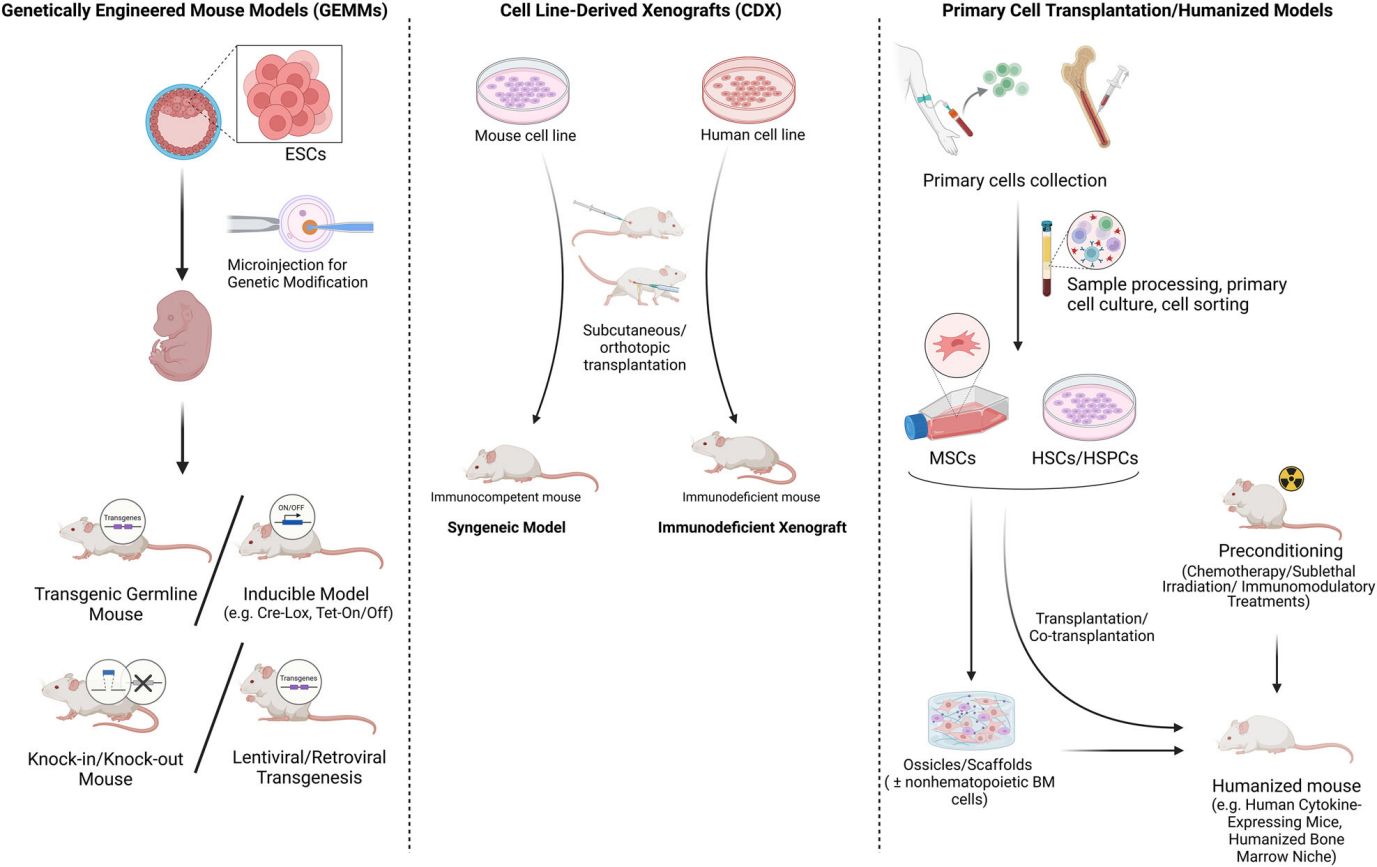
Strategies for developing mouse models of MDS. The left column illustrates genetically engineered mouse models (GEMMs), where MDS-related genetic modifications are introduced using transgenic, knock-in/knock-out, or inducible systems. The middle column represents cell line-derived xenografts (CDX), where mouse or human cell lines are transplanted into immunocompetent or immunodeficient mice, respectively. The right column depicts primary cell transplantation/humanized models, where primary human hematopoietic with or without stromal cells are transplanted into preconditioned mice to create a more physiologically relevant microenvironment for studying MDS. Created with BioRender.com.

The molecular heterogeneity of MDS reinforces the need for model systems that can represent the human disease pathogenesis. So, genetically engineered mouse models (GEMMs) have been generated to incorporate specific driver mutations, and can offer insight into disease mechanisms in an immune-competent system. A summary of mutation-specific mouse models and their correspondence to clinical MDS subtypes is provided in Table 2. On the other hand, patient derived xenograft (PDX) models can accurately reproduce the biological characteristics of the disease and facilitate the analysis of primary MDS cells in a murine environment. In addition, PDX models are particularly valuable tools to investigate therapeutic responses. The specific relevance of these models depends on their capacity to maintain clonal hierarchy, differentiation biases, and responsiveness to therapeutic interventions [25]. Figure 1 illustrates three key strategies for developing mouse models of MDS.

**Table 2:** Mutation-specific mouse models and their association with MDS subtypes.

Mutation	Clinical subtypes	Mouse models	References
SF3B1K700E	- MDS with low blasts and SF3B1 mutation - MDS with ring sideroblasts (MDS-RS) - MDS-RS with thrombocytosis (MDS/MPN-RS-T)	- Sf3b1-K700E knock-in: Mimics splicing defects, impaired erythropoiesis, progressive anemia but lacks complete ring sideroblast formation [93]. - Sf3b1^flox-K700E^/+ (Mx1-Cre): Conditional model with reduced hemoglobin and ineffective erythropoiesis. - Sf3b1^+/−^ heterozygous: Dyserythropoiesis, occasional ring sideroblasts in BM.	[22, 93, 125, 126]
SRSF2 ^P95H^	- MDS with multilineage dysplasia - MDS with excess blasts (RAEB-1/2) - CMML	- Srsf2^P95H^ knock-in: Macrocytic anemia, leukopenia, multilineage dysplasia, clonal HSC expansion; progression to AML in compound models.	[127–130]
TP53 mutations	- MDS with TP53 mutation (bi-allelic/multi-hit) - Therapy-related MDS - MDS with complex karyotype	- TP53 knockout or mutant knock-in mice ( ± cooperating mutations like NHD13 or RUNX1): Genomic instability, cytopenias, AML progression, therapy resistance.	[131–134]
TET2 and DNMT3A loss	- MDS with multilineage dysplasia - CMML - Clonal hematopoiesis (CHIP, precursor state)	- Tet2 or Dnmt3a knockout: HSC expansion, impaired differentiation, multilineage dysplasia, myeloid skewing; enhanced leukemogenesis with co-mutations. -Tet2 and Zrsr2 double mutant mice.	[105, 135, 136]
NRAS/KRAS mutations	- MDS with excess blasts (RAEB) - JMML - MDS/MPN overlap	- Nras^G12D^ or Kras^G12D^ knock-in: myeloproliferation, monocytosis, dysplasia, progression to AML; hyperactive RAS phenotype.	[111, 137–139]
NUP98-HOXD13 fusion	- Therapy-related MDS (t-MDS) - RCMD - MDS/AML transformation	- NHD13 transgenic (Vav1 promoter): anemia, neutropenia, dysplasia, macrocytosis, AML progression.	[91, 140–143]
ASXL1	- MDS with multilineage dysplasia - RAEB - High-risk MDS	- Asxl1 knockout or mutant mice: cytopenias, dysplasia, apoptosis, leukemic progression; enhanced with co-mutations.	[144, 145]
RUNX1	- RAEB-1/2 - Therapy-related MDS - MDS/AML with multilineage dysplasia	- Retroviral transduction (D171N, S291fs): long-latency MDS/AML, requires cooperating hits (e.g., *Evi1*). - Runx1^Δ/Δ^ (Mx1-Cre): anemia, trilineage dysplasia, rapid progression in *Mll-PTD* background.	[146]
U2AF1	- MDS with multilineage dysplasia - RAEB	- U2af1^S34F^ knock-in: impaired hematopoiesis, mild dysplasia, splicing defects.	[147]
EZH2	- MDS/MPN overlap - High-risk MDS - MDS with excess blasts	- Ezh2 conditional knockout: impaired differentiation, dysplasia, leukemic progression; cooperates with other mutations.	[148, 149]

Mapping of genetic alterations to specific mouse models and disease phenotypes reflecting various MDS subtypes.

## Genetically engineered immunodeficient models

### Foundational models: NSG and BRG mice

NOD/SCID/IL2rγ−/− (NSG) mice, characterized by the IL2rg null and Prkdc^scid^ mutations, remain the gold standard for human HSC engraftment due to their T, B, and NK cell deficiencies [26]. Their NOD genetic background improves compatibility with human CD47-SIRPα interactions, reducing macrophage-mediated clearance of engrafted cells compared with BRG mice [27, 28]. This allows human HSC engraftment levels to reach 60% in bone marrow by 16 weeks post-transplantation [29].

However, a significant limitation of NSG mice is their limited support for myeloid engraftment and differentiation, which complicates the modeling of MDS that frequently include erythroid and megakaryocytic dysplasia. As a result, derivative strains like NSG-SGM3 and NOG-EXL were designed to express human cytokines including GM-CSF and IL-3 in order improve myeloid and erythroid differentiation [1].

### CD3E humanized mice

While PDX models primarily focus on preserving tumor heterogeneity for cancer research, CD3E humanized mice provide a platform for detailed investigations into T cell receptor signaling and therapeutic antibody interactions. This model facilitates the study of T-cell interactions and responses to therapies targeting specific antigens, such as CD20 or CD19 [30].

CD3E humanized mice (e.g., BALBc-hCD3EDG) enable precise evaluation of T-cell-engaging therapies by preserving human CD3ε/δ/γ heterodimers critical for TCR signaling [31, 32]. This is particularly relevant to MDS, where immune dysregulation manifests as skewed regulatory T-cell (Treg) ratios and exhausted cytotoxic T cells [33, 34]. In CD3E models, anti-human thymocyte globulin (ATG) selectively depletes conventional T cells while sparing Tregs [34], recapitulating the immunosuppressive Treg dominance observed in significant number of MDS cases [35, 36].

A clinically relevant example is vibecotamab, a CD3–CD123 bispecific antibody, which achieved a 68% clinical benefit rate in MDS/CMML after failure of hypomethylating agents in a Phase II study [37]. In CD3E-humanized mouse models such as BALB/c-hCD3EDG, T-cell subpopulation distributions, including the CD4:CD8 ratio, are maintained at levels closely resembling wild-type mice, ensuring physiologically relevant immune responses [30].

To date, there is no direct mechanistic data to validate these models for MDS, but studies in analogous humanized and CD123-targeting systems show T-cell activation, IFN-γ release [38], and preferential targeting of CD123⁺ blasts over normal CD34⁺ progenitors [39]. These findings validate the model’s utility for studying checkpoint inhibitor responses and bispecific antibody efficacy in MDS-associated immune evasion.

CD3E humanized mice are developed by replacing the murine CD3E protein with its human version using gene targeting techniques. This modification allows T cells in these mice to effectively interact with human-specific antibodies, facilitating studies on T-cell activation and proliferation. These mice have normal thymic development and can be activated by crosslinking with anti-human CD3E antibodies, similar to wild-type T cells [40]. These models bind human CD3E antibodies and transduce the necessary signals for T-cell activation, making them particularly useful for screening anti-human CD3E antibodies and evaluating drugs that target the human CD3 signaling pathway [41]. Moreover, studies indicate that the distribution of lymphocyte subpopulations in CD3E humanized mice is aligned with wild-type mice, suggesting that introducing human CD3E does not affect overall immune cell functionality [30].

By enabling human-specific CD3E interactions, these models can be useful for testing immunotherapies, screening novel T cell-engaging and antibody therapies and investigating T cell-mediated immune responses.

### Cytokine-humanized models

One important challenge in modeling MDS is cytokine dependency since murine cytokines show minimal cross-reactivity with human hematopoietic cells, limiting the efficiency of human hematopoiesis in patient-derived xenograft (PDX) models [42]. The changes in the microenvironmental interactions between murine and human hematopoietic systems can influence disease progression since mouse stromal and immunological components do not perfectly match the human bone marrow niche [43]. Also, genetic background contributes to variations in disease progression, as some mutations may have distinct functional effects in mice compared to human hematopoietic cells [44].

Cytokine-humanized mouse models integrate human cytokines to augment the proliferation of donor-derived human cells and the development of the immune system. In contrast to traditional models, these have distinct cytokine expression profiles that influence their capacity to support diverse hematopoietic lineages using factors such SCF, CSF-1, GM-CSF, IL-3, IL-6, IL-7, and IL-15 [45].

As summarized in Table 3, selecting an appropriate humanized mouse model depends on the specific research objectives and the hematopoietic lineage under investigation.

**Table 3:** The key characteristics of the commonly used cytokine-humanized models.

Mouse model	Cytokines expressed	Advantages	Limitations	References
MISTRG	TPO, IL-3, GM-CSF, M-CSF	Supports multilineage differentiation including myeloid cells; suitable for studying MDS. Efficient engraftment of human hematopoietic cells.	Complex genetic modifications; potential immune drift. Anemia due to phagocytosis of mouse red blood cells by human macrophages.	[13, 82]
NSG-SGM3	IL-3, GM-CSF, SCF	Improved myeloid cell differentiation, suitable for MDS studies.	Severe anemia, graft exhaustion over time.	[150]
MITRG	IL-3, GM-CSF, TPO, SCF	Optimized for erythroid and myeloid differentiation, MDS studies.	Limited in complete immune system reconstitution.	[46, 151]
hIL-7 NOG	IL-7	Improves T-cell development, relevant for immunotherapy research.	Limited direct evidence, studies mostly on NSG-W41.	[56]

Comparison of major humanized strains (e.g., NSG-SGM3, MISTRG, NOG-EXL) regarding cytokine support, lineage reconstitution, and MDS applicability.

The differential expression of human cytokines in these strains demonstrates the importance of species-specific cytokine support in promoting efficient engraftment. Murine cytokines present suboptimal interactions with human hematopoietic cells, so there is the need to introduce human-specific cytokines that support the survival, proliferation, and differentiation of transplanted hematopoietic stem/progenitor cells (HSPCs) [45].

This is particularly relevant in MISTRG and MITRG mice, where human TPO, IL-3, GM-CSF, G-CSF, and SCF collectively promote the expansion of both myeloid and erythroid lineages, making these models ideal for studies of MDS progression [46]. This combination improves the survival of human HSCs and facilitates the development of functional macrophages, granulocytes, and NK cells. This expression pattern makes MISTRG highly effective for studying disorders that involve both myeloid and erythroid lineages, including MDS [47].

Similarly, NSG-SGM3 mice, which express human IL-3, GM-CSF, and SCF, have demonstrated improved myeloid cell differentiation, making them useful for investigating hematopoietic malignancies [29]. Still, their utility is limited by issues such as severe anemia and graft exhaustion over time [48]. Moreover, studies show that NSG-SGM3 mice are incapable of supporting MDS grafts long-term, with 100% of low-risk and 75% of high-risk MDS xenografts progressively losing engraftment viability after 24 weeks. This reduction is associated with insufficient support for lineage-specific differentiation processes, especially the TPO-mediated maturation of megakaryocytes, which is absent in existing NSG-derived models [1, 49].

In contrast, as mentioned, MISTRG have an optimized cytokine expression that facilitates both myeloid and erythroid lineage differentiation, by incorporating human TPO, making this strain of mice highly suitable for studying MDS [50].

In 2024, a study introduced MISTRG6kitW41 (M6k) as an improved cytokine-humanized PDX model for MDS research. By incorporating the c-kit W41/W41 mutation, M6k improves HSC engraftment without the need of irradiation preconditioning, and outperforms conventional MISTRG models, particularly in erythroid and megakaryocytic differentiation. At the molecular level, M6k preserves the genetic and transcriptomic profiles of patient-derived MDS samples and retains mutations such as *SRSF2, U2AF1, TET2, RUNX1, IDH2, ASXL1*. scRNA-seq analysis confirmed that CD34 + MDS cells in M6k mice closely showed a high similarity with the patient-derived equivalents. The model demonstrates stable huCD45+ and CD34+ engraftment in bone marrow and peripheral blood, supporting long-term disease simulation and replicating secondary AML progression. While NSG-SGM3 supports human hematopoiesis via transgenic cytokine expression, M6k enhances engraftment by impairing host stem cells through a c-kit mutation, promoting stable erythroid and megakaryocytic development [10].

The NOGW-EXL model represents an alternative to NSG-SGM3 for studies requiring sustained human hematopoiesis. Although not specifically developed for MDS, it can address some limitations of NSG-SGM3, such as anemia and graft exhaustion. This model carries a c-kit W41 mutation, which improves stem cell maintenance, and transgenically expresses human IL-3 and GM-CSF, supporting myeloid differentiation. Also, this mutation facilitates the engraftment of HSCs without requiring irradiation. Compared to NSG-SGM3, NOGW-EXL demonstrates improved long-term engraftment stability, particularly for granulocytes and megakaryocytes, making it a suitable alternative for preclinical research on MDS and related disorders [51, 52].

For example, in a study investigating alternative models to NSG-SGM3 for hematological malignancies, researchers demonstrated that NOG-EXL mice present a more controlled myeloid differentiation program, reducing excessive cytokine-driven activation. At molecular level, NOG-EXL mice presented more balanced activation of myeloid pathways, with reduced eosinophilic infiltrates and a lower incidence of mast cell hyperplasia compared to NSG-SGM3. Also, histological assessments showed that tissue infiltration by activated macrophages, which is prominent in NSG-SGM3, is significantly expressed in NOG-EXL, suggesting a more stable immune environment [48].

While cytokine-humanized models primarily support hematopoiesis, the Bone Marrow–Liver–Thymus (BLT) model is suitable for studying human immune function. BLT mice are generated by transplanting human fetal liver and thymus tissues under the kidney capsule of immunodeficient mice, followed by intravenous injection of CD34+ hematopoietic stem cells [53]. As a result, they develop a functional human adaptive immune system, with mature T and B cells, dendritic cells and macrophages. Their capacity to support proper thymic selection of human T cells makes them relevant for studies on immune responses, immune modulation in hematological diseases, and preclinical evaluation of immunotherapeutic strategies such as immune checkpoint inhibitors and CAR-T cell therapy [54]. Compared to cytokine-humanized models, which primarily support specific hematopoietic lineages, BLT mice provide a more complex immune system model, allowing for investigations into host-tumor interactions and immune dysregulation in MDS [55].

Additionally, hIL-7 NOG mice have been studied for their role in improving T-cell development, direct evidence on their use in hematological disease modeling is limited. For example, transgenic expression of hIL-7 under murine regulatory elements in NSGW41 mice has been shown to improve human T cell expansion following CD34+ hematopoietic stem cell transplantation [56]. These findings indicate that incorporating hIL-7 into humanized models can create a more physiologically relevant system for studying immune cell interactions and evaluating immunotherapeutic approaches. Direct evidence on hIL-7 NOG mice is limited but studies have shown that transgenic expression of human IL-7 in NSGW41 mice promotes the expansion of functional human T cells following CD34+ hematopoietic stem cell transplantation [56, 57]. Moreover, the administration of human IL-7 in vivo leads to a sustained increase in T-cell numbers [58].

## Patient-derived xenograft (PDX) in MDS research

MDS research has advanced through the use of PDX models, which are generated by transplanting primary human MDS cells, often mononuclear cells into immunodeficient mice. Unlike conventional murine models, such as GEMMs, PDXs preserve the genetic and phenotypic heterogeneity of the original disease, better reflecting its complexity [59].

### Co-transplantation models: NSG, NSG-SGM3

Multiple research groups have attempted to co-engraft MSCs and MDS cells to promote leukemic stem cell myeloid differentiation and persistence. In one study, researchers co-transplanted 10⁴ CD34 + MDS-SCs with MSCs into gelatin-based porous scaffolds implanted in NSG-SGM3 immunodeficient mice, achieving long-term engraftment rates ranging from 0.2% to 86% hCD45+ cells, with 82% of cases reaching ≥20% hCD45+ cells after 12 to 18 weeks. The engrafted cells preserved key MDS-associated mutations, including *SF3B1, DNMT3A, SRSF2*, and *TET2* [7].

In another study, transplanted 5 × 10⁵ CD34+ HSPCs and 1.5 × 10⁶ autologous MSCs from MDS patients into NSG mice using direct intramedullary injection, achieving 100% engraftment across patient samples. Human chimerism levels ranged from 59.7% to 0.0175% hCD45+ cells after 6 months, with secondary transplantation of 1 × 10⁵ CD34+ cells without MSCs maintaining 53.33% chimerism, demonstrating the long-term self-renewal capacity of MDS-initiating cells. Genetic analysis confirmed the stability of key mutations, including *TP53, SF3B1, RUNX1*, and *KIT*, across both primary and secondary recipients [9].

Some studies indicate improved engraftment with MSC co-injection, but others show minimal or no long-term advantage. Krevvata et al. examined the engraftment of MDS cells in NSG and NSG-SGM3 strains, showing that MSC co-injection provided a minimal influence on engraftment levels. MSCs offered temporary support to MDS cells, and the presence of MSCs did not substantially modify the clonal structure of the engrafted cells [8]. Rouault-Pierre et al. noted that MSC co-injection resulted in 100% engraftment in NSG and NSG-SGM3 mice but human chimerism levels showed considerable variability, spanning from 59.7% to 0.0175% hCD45+ cells after 6 months. The secondary transplantation of 1 × 10⁵ CD34+ cells without MSCs resulted in 53.33% chimerism, which indicates that MSCs are not critical for the maintenance of long-term hematopoiesis [6].

## Preconditioning strategies for improved engraftment

Recent studies have optimized PDX models to improve the representation of low-risk MDS, characterized by low CD34+ blast counts and limited clonal dominance [11]. One important advancement in this field has been the optimization of preconditioning strategies to improve human hematopoietic engraftment in immunodeficient mice. A comparative summary of preconditioning strategies and their impact on engraftment efficiency across commonly used mouse strains is presented in Table 4. Traditionally, sublethal irradiation with doses varying from 200–375 cGy has been used to suppress murine hematopoiesis, creating space for transplanted human MDS cells [60–62]. In some cases, irradiation alone has been insufficient to achieve optimal engraftment, requiring the implementation of alternative or complementary methodologies.

**Table 4:** Preconditioning responses and engraftment potential across common mouse strains in MDS.

Strain	Immune status	Preconditioning response	Best used for	References
NSG	Highly immunodeficient (lacks T, B, and NK cells)	Requires irradiation or busulfan for optimal engraftment	General human hematopoietic engraftment studies	[26]
NSG-S	NSG with improved macrophage depletion	More effective macrophage depletion with clodronate liposomes	Enhanced erythroid precursor survival and engraftment	[10]
NSG-SGM3	NSG expressing human cytokines (SCF, GM-CSF, IL)	Supports myeloid differentiation due to cytokine expression; may develop severe, fatal myeloid activation syndrome	Myeloid differentiation and transplantation studies; limited by shorter lifespan due to myeloid activation syndrome	[48, 152]
NOG	Similar to NSG, but distinct IL-2 receptor gamma chain mutation	Comparable to NSG; requires irradiation or busulfan for engraftment	Alternative to NSG with similar applications	[153]
NRG	NOD-Rag1-null IL2Rγ-null; similar to NSG but with different immune defects	Comparable to NSG; potential differences in radiation sensitivity	Studies requiring NSG-like immunodeficiency	[154]
NOG-EXL	NOG expressing human GM-CSF and IL-3	Provides stable engraftment with extended lifespan; less severe myeloid activation syndrome compared to NSG-SGM3	Long-term studies involving myeloid lineage cells	[52]

Evaluation of irradiation or chemotherapy preconditioning and resulting human hematopoietic cell engraftment levels in different murine backgrounds.

To address the challenge of low-risk MDS modeling, Teodorescu et al. used NSG-S mice preconditioned with clodronate liposomes and low-dose radiation to model low-risk MDS, improving engraftment efficiency. Clodronate liposomes, administered intraperitoneally at 100 µL per animal 2 days before irradiation, depleted murine macrophages by up to 97% in bone marrow and over 99% in spleen, inhibiting human erythroid precursor clearance. Before intravenous transplantation of human CD34+ cells, mice received 2 Gy radiation on day 0 to inhibit residual mouse hematopoiesis. This method effectively preserved the genetic profile of low-risk MDS, including mutations such as *SF3B1, ZRSR2*, and *ASXL1*, while supporting long-term hematopoiesis [11].

Another strategy is the use of ultra-low dose irradiation (0.5 Gy) as a preconditioning method. A recent study, showed that this approach facilitates engraftment while preserving the bone marrow microenvironment. The low dose did not compromise the vascular barrier (increase vascular permeability). In general, an irradiation threshold of 2 Gy has been reported to induce secretion of pro-inflammatory mediators, degradation of endothelial junctions, and an increase in vascular permeability and allowed long-term engraftment compared to non-conditioned host animals [63].

Antypiuk et al. developed an alternative preconditioning method by targeting iron metabolism in CD45.2 + MDS mice. Using *TMPRSS6* silencing via GalNAc-conjugated siRNA in CD45.2 + MDS mice they increased hepcidin levels, reducing conditioning-induced Non-Transferrin Bound Iron and improving donor HSPC engraftment after BMT. Their results suggest that iron restriction improved transplantation outcomes by reducing chemotherapy-induced toxicity [64].

Another option is busulfan, a chemotherapeutic drug that eliminates host hematopoietic cells without requiring radiation exposure. Research has shown that administering busulfan at 20 mg/kg intraperitoneally may produce similar levels of hematopoietic suppression in mice [65, 66]. However, busulfan can induce significant toxic effects. Doses ≥25 mg/kg/day in NSG mice can result in increased severity of clinical signs and mortalities between Days 1 and 25 [67]. Also, busulfan can also induce toxic effects on the epididymis and cause long-term morphological damage to sperm, potentially leading to permanent sterilization [68].

**Fig. 2:**
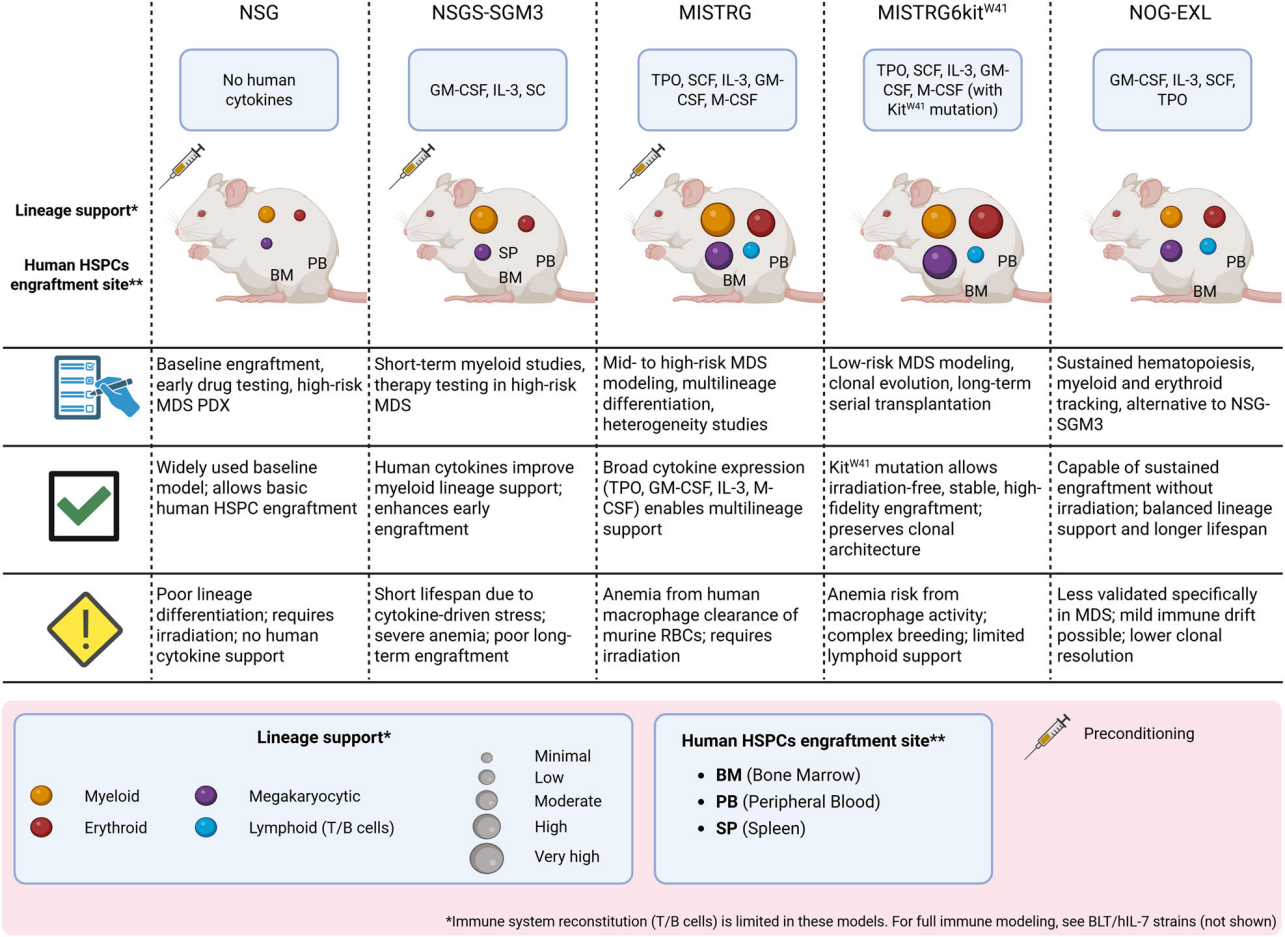
Comparative features of humanized mouse models in MDS research. Five immunodeficient mouse models commonly utilized for MDS research (NSG, NSGS-SGM3, MISTRG, MISTRG KitW41, and NOG-EXL) are presented. Each mouse model is depicted with key human cytokines they express, lineage support capacity (myeloid, erythroid, megakaryocytic, and lymphoid lineages), and sites of human hematopoietic stem and progenitor cell (HSPC) engraftment. Color and size-coded circles illustrate the extent of lineage differentiation support provided by each model, ranging from minimal to very high. Additionally, the figure summarizes key characteristics and commonly associated applications of each model (clipboard symbol), as well as their advantages in supporting human hematopoiesis (checkmark symbol) and their experimental limitations (exclamation mark symbol). Created with BioRender.com.

Figure 2 provides a comparative overview of commonly used immunodeficient mouse models in MDS research, including their cytokine expression profiles, lineage support capacities, engraftment sites, and key experimental features.

## Alternative genetically diverse models

In addition to humanized models, genetically diverse mouse strains have been also developed to investigate the impact of host genetics on MDS pathogenesis and disease heterogeneity. To address genetic diversity limitations in mouse modeling, The Collaborative Cross (CC) mouse population has been developed, which captures over 90% of the genetic variation in mice, and has been proposed as an alternative for more representative disease modeling [69]. Compared to traditional inbred strains, CC mice facilitate the study of genetic factors that influence disease susceptibility, clonal evolution and treatment response variability [70]. Among CC strains, the JUN mouse strain has been identified as a candidate due to its spontaneous development of MDS-like symptoms, including cytopenia and abnormal differentiation of bone marrow progenitor cells. These strains are important for investigating host-specific genetic contributions to MDS, but their translational utility is limited, as murine hematopoietic and immune systems differ significantly from human counterparts. While CC and JUN mice allow for the study of genetic susceptibility and environmental influences in MDS, humanized models are the primary platform for translational research, particularly in drug development and immunotherapy [71].

## Preclinical modeling using cell line-derived xenografts

Most MDS cell lines do not successfully develop stable xenografts due to their dependency on the microenvironment, and insufficient murine cytokine support. Compared to AML cell lines, MDS cells have a limited proliferative capacity and require exogenous cytokines, like IL-3, GM-CSF, and SCF, which is limiting in vivo studies [8]. For example, MDS-L has two subclones, MDS-L-2007 and MDS-LGF, each with distinct IL-3 dependency and proliferation capacity [72]. MDS-LGF proliferates with minimal IL-3 (1 ng/mL) and achieves higher engraftment rates, while MDS-L-2007 needs higher supplementation of IL-3 (100 ng/mL), which reduces the in vivo applicability [8].

Several studies have classified MDS cell lines into three distinct groups: false/non-malignant cell lines, malignant cell lines in the leukemic phase and valid MDS cell lines [1, 23].

As summarized in Table 5, only a subset of characterized MDS cell lines including M-TAT, MDS92, TER-3, and MDS-L serve as standardized experimental models for studying disease pathogenesis and therapeutic responses. These models have been validated through foundational studies, which demonstrate their functional attributes and genetic profiles, confirming their relevance in preclinical research.

**Table 5:** Summary of myelodysplastic syndrome cell lines: origins, lineage, and key features.

Cell line	Original disease	Lineage	Characteristics	References
M-TAT	RAEB-MDS	Erythroid or megakaryocytic	Isolated from peripheral blood of 3-years-old patient.	[155]
			Cytokine-dependent (EPO, GM-CSF, SCF, IL3).	
			Differentiates in response to EPO but shows reduced hemoglobin when GM-CSF is added.	
MDS92	RARS-MDS	Myeloid with megakaryocytic differentiation	Isolated from a bone marrow of 52-year-old male.	[72, 156]
			Presence of typical MDS aberrations such as 5q and 17p deletion, monosomy 7 and NRAS mutation.	
			Sensitive to cytokines such as GM-CSF and IL-3.	
TER-3	RAEB-MDS	Erythroid or megakaryocytic	Cytokine-dependent: G-CSF, GM-CSF, IL3, TPO, M-CSF, SCF.	[23, 157]
			TER-3 has surface markers that are strongly positive for myeloid, lymphoid, and megakaryocytic antigens, including CD15, CD19, and CD6.	
			Presence of typical MDS aberrations such as monosomy 7,20 and Y.	
MDS-L	MDS (often progressing to AML)	Myeloid	Blastic subline derived from MDS92.	[72, 158]
			A standard preclinical model for developing MDS therapies.	
			Sensitive to IL-3 cytokine.	
			H3-K27M mutation was detected in MDS-L.	
MDS-L-2007	MDS with leukemic transformation	Myeloid	MDS-L in the presence of IL-3 generated MDS-L-2007 (H3-K27M–mutant).	[158]
MDS-LGF	MDS with leukemic features	Myeloid	MDS-L in the absence of IL-3 generated MDS-LGF (H3-K27M–wild type).	[158]

Key properties of available MDS-derived cell lines used in experimental modeling, including lineage origin, mutational status, and in vivo utility.

## Diagnostic alignment: progress and pitfalls

The diagnosis of MDS in animal models requires criteria that balance translational relevance to human disease with species-specific biological differences. The Mouse Models of Human Cancers Consortium (MMHCC) established diagnostic guidelines to standardize the classification of murine hematopoietic neoplasms, addressing inconsistencies in preclinical MDS research. This system categorizes nonlymphoid hematopoietic neoplasms into four groups: nonlymphoid leukemias, hematopoietic sarcomas, myeloid dysplasias, and myeloid proliferations [14].

According to this system, the diagnosis of nonlymphoid leukemia requires the presence of ≥20% immature forms or blasts in the bone marrow or spleen. In contrast, myeloid dysplasias are characterized by cytopenias and abnormal differentiation, with the exclusion of cases exhibiting ≥20% blasts. These cytopenias include neutropenia, anemia, or thrombocytopenia without leukocytosis or erythrocytosis being present. Morphological abnormalities can further support this diagnosis. Dyserythropoiesis is recognized by megaloblastic changes, fragmented nuclei, or ringed sideroblasts, while dysgranulopoiesis is characterized by atypical nuclear and cytoplasmic maturation. Dysplastic megakaryocytes are marked by multiple distinct nuclei or micromegakaryocytes [73, 74]. Moreover, molecular tests could help to confirm the existence of a monoclonal population [75].

While these criteria reflect human diagnostic parameters, including cytopenias, dysplasia, and blast thresholds, they do not fully capture species-specific differences that limit translational accuracy. For example, in humans, bone marrow fat (BMF) constitutes ~70% of marrow volume in adults and regulates HSC behavior through adipocyte-derived factors like leptin and adiponectin. With aging, BMF expansion correlates with reduced hematopoiesis which can drive MDS progression [14].

In contrast, murine marrow contains <5% BMF, and they may fail to capture for example adipokine-mediated inflammation, like elevated TNF-α and IL-6 which are representative for human MDS [43].

Also, while genetically engineered models can replicate individual mutations like *TP53* or *SF3B1*, they can fail to capture the clonal complexity of human MDS, where the sequential accumulation of mutations such as *TET2, ASXL1*, and splicing factors drives disease progression [14].

In terms of validation practices, studies show that only 12% of MDS mouse studies report randomization, while less than 5% implement blinding. This aspect could introduce bias and reduce the relevance of the results and therefore limiting the translational applicability [76]. To improve the protocol standardization and crosslab reproducibility, the preregistration of preclinical protocols in platforms like preclinical.eu or animalstudyregistry.org could reduce selective reporting [77].

## Emerging trends and future directions

The development of humanized mouse models has transformed and expanded our capacity to study MDS, however critical limitations remain to be addressed, limiting their ability to fully reproduce the complexity of human disease.

The Minimal Information for Standardization of Humanized Mice (MISHUM) initiative points out the need for community-driven, standardized reporting guidelines that improve quality and reproducibility in these models; still these frameworks have not been systematically implemented in MDS research due to persistent challenges in protocol alignment and data standardization [78].

Other guidelines like the ARRIVE 2.0 [79] and PREPARE guidelines [80] provide foundational principles for transparent reporting and precise experimental design, but their application to MDS-specific studies is inconsistent. Although standardization of MDS mouse models is achievable in principle, its practical implementation remains complex due to the need for precise, shared databases and consensus on experimental design, which are currently lacking in most research settings. As discussed in recent reviews, the heterogeneous nature of MDS and the variability in model systems, and insufficient reporting of critical parameters present significant challenges to the development of universally accepted standards [78].

In an overview of 31 systematic reviews, Hirst et al. reported that only 29% of animal studies using models such as mice documented randomization, 15% reported allocation concealment, and 35% reported blinded outcome assessment. Lack of randomization was significantly associated with exaggerated effect sizes. These results indicate substantial methodological deficiencies in animal research, which undermine the reliability and translational relevance of murine models, including those applied in MDS [81].

While cytokine supplementation (e.g., SCF, GM-CSF, IL-3) and niche-specific conditioning (e.g., c-kit targeting) have improved human hematopoietic cell engraftment in murine models, they still fail to replicate the full complexity of BME [82]. A major challenge is the rapid clearance of mature human blood cells, like platelets and erythrocytes, by the host immune system. Current strategies, like anti-CD117 antibodies or transient immune suppression, offer only partial solutions. Advancing humanized models is important to simulate better MDS and improve long-term engraftment [83].

Most current MDS research is focused on high-frequency mutations (*SF3B1*, *TP53*) while omitting rarer variants that have distinct clinical impacts. *SF3B1* mutations, present in 31% of MDS cases, are studied intensively due to their association with favorable outcomes in ring sideroblast subtypes [84]. Similarly, *TP53* mutations ( ~ 25% prevalence) are extensively studied for their link to aggressive disease and treatment resistance [85]. While mouse models have advanced our understanding of common MDS-associated mutations like *SF3B1* and *TP53*, the field requires models that capture rare but clinically significant variants such as *BCOR* and *STAG2* [86].

While advanced murine models of MDS have started to cover immune evasion strategies, like Treg expansion or CD8 + T-cell exhaustion, there is a gap in understanding the causality and mutation-specific dynamics [87, 88].

For example, studies on transgenic TRAF6-overexpressing mice show that MDS HSPCs can adapt to inflammation by upregulating *A20*. This favors the survival of MDS HSPCs while normal HSPCs are affected by inflammatory stress [89]. However, these murine models are mostly used to study late stages disease, like NUP98-HOXD13 or S100A9 transgenic mice, which replicate advanced MDS phenotype, but offer insights about early clonal competition or pre-malignant stages [90, 91].

The next generation of MDS models should move beyond studying high-frequency mutations and late-stage disease to incorporate the full spectrum of genetic, immune, and stromal interactions. We anticipate that these improvements would ultimately lead to more efficient preclinical drug development and better personalized treatment strategies.
